# Invasive versus Non Invasive Methods Applied to Mummy Research: Will This Controversy Ever Be Solved?

**DOI:** 10.1155/2015/192829

**Published:** 2015-08-06

**Authors:** Despina Moissidou, Jasmine Day, Dong Hoon Shin, Raffaella Bianucci

**Affiliations:** ^1^Department of Histology and Embryology, Medical School, National Kapodistrian University of Athens, 75 M. Asias Street, 11527 Athens, Greece; ^2^The Ancient Egypt Society of Western Australia Inc., P.O. Box 103, Ballajura, WA 6066, Australia; ^3^Division of Paleopathology, Institute of Forensic Science, Seoul National University College of Medicine, Seoul 110-799, Republic of Korea; ^4^Department of Public Health and Paediatric Sciences, Legal Medicine Section, University of Turin, Corso Galileo Galilei 22, 10126 Turin, Italy; ^5^Center for Ecological and Evolutionary Synthesis (CEES), Department of Biosciences, University of Oslo, P.O. Box 1066, Blindern, 0316 Oslo, Norway; ^6^Anthropologie Bioculturelle, Droit, Ethique et Santé, Faculté de Médecine-Nord, Aix-Marseille Université, 15 boulevard Pierre Dramard, 13344 Marseille Cedex 15, France

## Abstract

Advances in the application of non invasive techniques to mummified remains have shed new light on past diseases. The virtual inspection of a corpse, which has almost completely replaced classical autopsy, has proven to be important especially when dealing with valuable museum specimens. In spite of some very rewarding results, there are still many open questions. Non invasive techniques provide information on hard and soft tissue pathologies and allow information to be gleaned concerning mummification practices (e.g., ancient Egyptian artificial mummification). Nevertheless, there are other fields of mummy studies in which the results provided by non invasive techniques are not always self-explanatory. Reliance exclusively upon virtual diagnoses can sometimes lead to inconclusive and misleading interpretations. On the other hand, several types of investigation (e.g., histology, paleomicrobiology, and biochemistry), although minimally invasive, require direct contact with the bodies and, for this reason, are often avoided, particularly by museum curators. Here we present an overview of the non invasive and invasive techniques currently used in mummy studies and propose an approach that might solve these conflicts.

## 1. Introduction

Mummies represent a unique source of information about past diseases and their evolution. The question as to how to best maintain the integrity of archaeological and anthropological specimens in the course of examining this evidence has been a major cause for dispute among scholars.

The advent of non invasive techniques (e.g., X-ray, CAT scanning, and MRI) for examining mummified remains has been a breakthrough in paleopathology as retrospective diagnoses can now be achieved without dissection.

However, mainly because of the structural differences between modern and ancient soft tissues, the efficiency of non invasive techniques has been questioned repeatedly. Many scholars insist that an accurate diagnosis can be correctly made only through direct examination of the corpse (i.e., autopsy, endoscopy). However, this approach creates concern among curators and archaeologists.

Here we address the debate from a broader perspective considering the advantages and disadvantages of both invasive and non invasive methods and propose the creation of an examination protocol for the analysis of ancient mummified remains based upon strict scientific and ethical criteria.

## 2. The Use of Non Invasive Techniques in Paleopathology

A new era in mummy studies began when the first group of Egyptian mummies was subjected to computed tomography (CT) in 1979 [1]. Scientists were given the opportunity to inspect the ancient Egyptians' bodies without resorting to the use of invasive methods [2, 3]. Both hard and soft tissues could be differentiated from multiple textile layers and artifacts (amulets, death masks, or portraits) and their pathologies diagnosed.

Although diagenetic alterations of ancient tissues often generate interpretative biases, CT scans have allowed differentiation between tissue structures and embalming materials. Similarly, antemortem traumas could be distinguished from postmortem manipulations associated with the embalming process [4], generating greater knowledge about the ways in which ancient populations treated and preserved their dead.

Artificial mummification is the deliberate act of preservation of a body after death [5]. This practice is aimed at slowing and/or halting soft tissues' degradation [6]. Different types of treatments (e.g., evisceration, use of natron, and coating with complex mixtures with antibacterial and antiputrefactive properties) allowed long-term preservation of the Egyptian mummies [7, 8].

Apart from exceptional cases, in which some steps of the mummification procedure were documented (i.e., the coffin of Djedbastiuefankh, Pelizaeus Museum, Hildesheim, Late Period; the Rhind Magical Papyrus, ca. 200 BC; three papyri in Cairo, Durham Oriental and Louvre Museums, around 1st century AD), the Egyptians did not leave written or illustrated records of their mummification methods [5].

Gaps in direct evidence have, therefore, been filled with information derived from numerous written sources. Herodotus (5th century AD) provided the earliest written accounts of mummification (Book II of* The Histories*). This is coupled with the records of Diodorus Siculus (1st century BC) and further augmented by the writings of Porphyry (3rd century AD). These principal sources have long provided the basis of modern knowledge about Egyptian mummification techniques [9]. CT scans have thus helped scientists and Egyptologists to increase their knowledge, which had hitherto been biased by the cultural stereotyping of Egypt in classical sources. New and more detailed knowledge about the evolution of artificial mummification has emerged [10, 11].

Over the last decade, a new generation of CAT scanners with increased power of resolution has been released and virtual autopsy has become one of the basic steps in any scientific investigation of mummified remains [12]. Visualization technology is an efficient tool in hard and soft tissue paleopathology [13]. Dental diseases (e.g., severe teeth abrasion, carious lesions, cists/abscesses, inflammation, and tooth loss) [14] and many degenerative disorders (e.g., rheumatoid arthritis of the Iceman [15], anthropo-paleopathological in Egyptian mummies [16], bone and soft tissue malignant tumours and/or soft tissue adenomas [17], or atherosclerosis [18, 19]) can now be diagnosed.

The latest developments in CT resolution (MicroCT) have even enabled the observation of architectural structures of bones [20].

Variations in wavelength radiation or use of Terahertz imaging have also been applied to mummified remains. Depending on the degree of hydration of a mummified corpse, this technique enables scientists to distinguish between features of soft tissues or bone and various artifacts, identifying objects [21] wrapped within textiles.

MRI (magnetic resonance imaging) has been similarly advantageous, especially in the study of hydrated mummies (i.e., bog bodies, South Korean mummies) [22]. MRI application on a 17th century Korean body showed unique clear organ structures, which could not be visualized by CT [23]. Less satisfactory results were obtained from MRI applied to dehydrated and embalmed bodies (i.e., Egyptian mummies) [24–26].

Despite the increased use of non invasive techniques, scholars still debate whether virtual inspection should be regarded as the “gold standard” in mummy studies or not.

In general, the CT methodological reference standards applied to the study of ancient remains are those determined from living patients [27]. Any kind of modification to CT scanning methodology (e.g., slice thickness or the introduction of other non invasive methods such as ionizing radiation on mummified cells) is still on an experimental level and is applied mainly in pilot studies of uncertain potential [28].

As a result of the differences between modern and ancient tissues and the absence of well-established methodological standards in mummy studies, misdiagnosis can occur. Gravitational force modifies both the morphology and location of the organs that elapses between burial and exhumation. Organs are displaced to the dorsal portion and their contraction is severe. While the difference in radiodensity is very important at a diagnostic level for living patients, radiodensity does not differ from organ to organ in mummies. Diagenetic processes can be misidentified as pathological conditions and vice versa [20]. To avoid misinterpretations, the complementary use of invasive methods (i.e., endoscopy, histology) is of utmost importance, either to support or to reject an initial diagnosis.

Another problem associated with the exclusive use of non invasive techniques is the lack of multidisciplinarity in teams involved in the interpretations of the data. Valid scientific research should include trained radiologists, whose experience lies mostly in diagnosing living patients, physical anthropologists or paleopathologists, and archaeologists who provide background information [4, 19, 22, 29].

To some extent concerning the scope of non invasive techniques applied to the study of ancient mummies generates confusion; therefore, the scientific purpose of non invasive methods often loses its meaning.

Museum curators and conservation experts usually prefer to resort to CT scanning in order to avoid specimen sampling and usually disregard the need for an overall anthropopaleopathological investigation. As a result, the broader use of non invasive techniques has become a fad, a spectacle misused by some scientists and curators for “infotainment” or advertising purposes. In many cases, the motivation for the employment of visual imaging is to take a curious glimpse inside a mummy [12] and perform animated 3D rendering for public display in exhibits rather than acquire sound scientific data.

## 3. The Role of Invasive Methods in Mummy Studies

Prior to recent advances in paleoradiology, invasive methods were the only available means of examining anthropological materials scientifically. Precious information about ancient lifestyles and diseases was acquired over decades, allowing scholars to gain a more profound historical and biological knowledge about populations of the past. The first invasive examination of ancient mummies began during the early 19th century, albeit as a form of public entertainment [2]. Many mummy unwrappings were carried out on the basis of mere curiosity and limited scientific knowledge. Therefore, many specimens were partially or completely destroyed.

Gradually, mummy autopsy became a more meticulous postmortem and provided scientists with information about both pathologies and possible causes of death [30].

Pioneering palaeopathologists adapted modern laboratory techniques to tiny mummified tissue biopsies in order to identify ancient tissue structures. They successfully diagnosed many diseases (e.g., tuberculosis, atherosclerosis, and parasitic diseases) [31–35].

Step by step, scientists have developed new methods to sample inner organ tissues, for example, endoscopy through natural orifices (i.e., mouth, nasal cavities, and use of forceps), and have progressively reduced the damage caused to mummies [36, 37].

Where endoscopes could not be introduced through natural or postmortem openings, a small perforation was made in the mummy's back [38], so that tissue samples could be taken for histological studies.

As in forensic pathology [39], microscopic examination of small tissue biopsies (0.7 × 0.7 cm) is a requisite to complement non invasive methods because it allows an initial diagnosis to be precisely confirmed or infirmed [17, 35, 40, 41].

Similar developments in gas chromatography/mass spectrometry (GC/MS), isotopic analysis, and synchrotron analysis of minimal amounts of mummy hair have provided remarkable information about the daily lives of ancient populations within various social classes [42–44] and detected chronic or acute exposure to heavy metals [45–47].

Advances in paleoimmunology [48–50] and paleomicrobiology through soft/hard tissue analysis and secretion swabs led to the retrospective diagnosis of several pathogens (e.g., salmonellosis, tuberculosis, malaria, human leishmaniasis, and Chagas disease) in mummies [51–67] and revealed some of their evolutionary patterns [68]. However, not all scientists agree that it is possible to recover ancient endogenous human and pathogenic DNAs from Egyptian mummies [69–71].

Sampling of small skin tissue biopsies (0.7 × 0.7 cm) and textiles (1 × 1 cm) proved to be a reliable method for assessing potential biodeterioration of a mummified body or its external contamination. Microorganism identification through cultivation and molecular techniques is extremely useful for conservation purposes and to minimize the risk of potential hazards to the public, especially when mummies are on display [72].

Biochemical investigations (a combination of gas chromatography-mass spectrometry, GC-MS, and thermal desorption/pyrolysis, TD/Py-GC-MS) applied to skin and textiles and to dental calculus provide a plethora of information concerning the recipes used in embalming procedures [73–77] and the diets of ancient populations [78, 79].

Nowadays, the use of invasive methods for examining mummies is widely regarded with skepticism. While some researchers consider autopsies unavoidable, many consider them a destructive procedure [80].

Full autopsy has often been performed mainly to see inside a mummy and take samples for experimental research rather than obtain confirmation of a disease tentatively identified via a non invasive method.

The archaeological value of a human/animal specimen must always be a primary concern, especially when it is on display. When mummies are completely wrapped, fully dressed, and accompanied by funerary equipment, the prospect of a full autopsy threatens their integrity [20]. Whereas CT imaging requires only careful transportation of the mummy, invasive examination is more complex but can potentially be performed in a manner that respects the integrity of the corpse [29].

Equally significant is the ethical issue concerning lack of respect for a human body. A mummy is a deceased person, not an artifact, and burial customs should not be ignored [81]. If this assumption is followed, no sampling or limited sampling should be allowed in order to respect the deceased, and it is equally true that presenting 3D virtual renderings of undressed dead bodies to a lay public also raises ethical concerns.

Questions concerning the analyses of anthropological remains have been raised in many countries and these call for a specific set of bioethical guidelines [82].

The extent of invasive examination to which ancient mummies should be subjected is the cause of much debate. In the absence of specific laboratory guidelines and protocols, there is a lack of consistency; this has allowed people without sufficient if any scientific background to decide how valuable samples should be investigated. In most countries, decisions rely mostly upon the protocols established by individual institutes, museums, or team supervisors. Decisions based upon such independent judgments may therefore vary from full autopsy permission to total prohibition of the use of any invasive technique.

## 4. Discussion and Conclusion

### 4.1. Is the Examination Method the Real Issue?

The controversy over the necessity of invasive versus non invasive techniques calls for some appropriate and standardized protocols to be applied to mummy research. The issue is not the effectiveness of invasive or non invasive studies but their suitability for mummy research, which at present does not consistently achieve scientific standards.

Firstly, the purposes of many studies are inconsistent. A mummy must be investigated in order to provide scholars with answers related to specific biological or historical questions. An investigation performed simply to observe a mummy macroscopically or microscopically is not useful and is not ethical. Curiously, only a limited number of studies focusing upon specific diseases or historical developments in funerary artifact types found upon mummies have been performed to date.

Secondly, artificial mummification techniques vary considerably according to environmental conditions and cultural practices. Various factors, temperature, humidity, soil acidity, and time, cause various cell system modifications; these can be pinpointed both through invasive and non invasive techniques. The state of preservation, fully intact or partially preserved, is significant even for mummies of similar type, which means that every mummy is a unique case.

Despite the numerous studies performed upon mummies, methodological consistency and scientific comparison are lacking. Validity of results cannot be cross-checked for the lack of comparative studies and when scientists from various disciplines collaborate in multidisciplinary studies, conflict of interests is not uncommon.

### 4.2. Mummy Research Guidelines: The Need for an International Ethical and Scientific Committee

The aim of this review is to show that the current controversy is mainly caused by a lack of internationally established guidelines in mummy research. This, in turn, calls for an international mummy research protocol to be instituted. Composed of scholars of high repute, whose integrity is widely recognized, a committee should reestablish a series of priorities in the study of mummified bodies.

Firstly, ethical issues should be considered [83]. Scientists need to pay respect to the funerary beliefs of the deceased. With advice from cultural anthropologists, ethnologists, and bioethicists, a specific protocol to approach each type of cultural/religious context should be designed.

Secondly, mummy studies should be allowed for scientific and educational purposes but not for business (i.e., public entertainment or commercial movies). The purpose of a given study, either medical or archaeological, should be disclosed before any kind of investigation is performed, its value being widely recognized by the scientific community. Similarly, as many neophytes approach the field without proper training, strict selection criteria should be applied.

Obviously, it is impossible to apply a rigid and inflexible scientific protocol to all mummy cohorts. While some universal principles and rules will apply, technical protocols will necessarily need to be adjusted depending upon the type of mummy (i.e., dry or hydrated) and its state of preservation (i.e., fully wrapped, intact, partially destroyed, etc.). Nonetheless, all parameters used for mummy investigations should be clearly detailed and results fully published.

More transparency should be demanded when genetic studies are released. Entire datasets should be published rather than selected sequences. This would enable other researchers to provide the scientific community with their own interpretations and critical assessments of the data. The absence of transparency through selective data publication only gives rise to accusations of secrecy that taint the name of science and reputation of the data.

Along with an international protocol for mummy investigations, the creation of a worldwide network of tissue banks would be an optimal solution. Scientists could be provided with samples for laboratory research without frequent examination of the original remains [84] and their research would generate a comparative database with which to enable more targeted scientific applications.

Mummies represent the most precious anthropological material with which ancient cultures have provided us. Since mummified bodies attract scientists from different fields, an international protocol is now essential and required urgently. This protocol should clearly answer three main questions: “Are we showing adequate respect to the corpse we are analyzing?”, “Which scientific hypothesis necessitates our study of mummified remains?”, and “Do we propose to study mummies for scientific/cultural purposes or for business?”

With the aim of creating a scientific committee and, subsequently, of promoting the standardisation of a bioethical protocol on mummified remains, the authors plan to organise a dedicated symposium within the next World Congress on Mummy Studies (Lima, July 27–30, 2016).
